# Malaria Elimination in Costa Rica: Changes in Treatment and Mass Drug Administration

**DOI:** 10.3390/microorganisms8070984

**Published:** 2020-06-30

**Authors:** Luis F. Chaves, John H. Huber, Obdulio Rojas Salas, Melissa Ramírez Rojas, Luis M. Romero, José M. Gutiérrez Alvarado, T. Alex Perkins, Monica Prado, Rodrigo Marín Rodríguez

**Affiliations:** 1 Vigilancia de la Salud, Ministerio de Salud, San José, San José 10123-1000, Apartado Postal, Costa Rica; melissa.ramirez@misalud.go.cr (M.R.R.); baduel72@gmail.com (J.M.G.A.); rodrigo.marin@misalud.go.cr (R.M.R.); 2 Department of Biological Sciences, University of Notre Dame, Notre Dame, IN 46556, USA; jhuber3@nd.edu (J.H.H.); taperkins@nd.edu (T.A.P.); 3 Programa Nacional de Manejo de Vectores, Región Huétar Norte, Ministerio de Salud, Muelle de San Carlos, Alajuela 21006, Código Postal, Costa Rica; obdulio.rojas@misalud.go.cr; 4 Departamento de Patología, Escuela de Medicina Veterinaria, Universidad Nacional, Heredia, Heredia 304-3000, Apartado Postal, Costa Rica; luis.romero.vega@una.cr; 5 Unidad de Investigación en *Plasmodium*, Centro de Investigación en Enfermedades Tropicales (CIET), Facultad de Microbiología, Universidad de Costa Rica, San Pedro, San José 11501-2060, Apartado Postal, Costa Rica; monica.pradoporras@ucr.ac.cr

**Keywords:** malaria, *Plasmodium vivax*, *Anopheles albimanus*, illegal mining, migration, Costa Rica

## Abstract

Costa Rica is a candidate to eliminate malaria by 2020. The remaining malaria transmission hotspots are located within the Huétar Norte Region (HNR), where 90% of the country’s 147 malaria cases have occurred since 2016, following a 33-month period without transmission. Here, we examine changes in transmission with the implementation of a supervised seven-day chloroquine and primaquine treatment (7DCPT). We also evaluate the impact of a focal mass drug administration (MDA) in January 2019 at Boca Arenal, the town in HNR reporting the greatest local transmission. We found that the change to a seven-day treatment protocol, from the prior five-day program, was associated with a 98% reduction in malaria transmission. The MDA helped to reduce transmission, keeping the basic reproduction number, *R_T_*, significantly below 1, for at least four months. However, following new imported cases from Nicaragua, autochthonous transmission resumed. Our results highlight the importance of appropriate treatment delivery to reduce malaria transmission, and the challenge that highly mobile populations, if their malaria is not treated, pose to regional elimination efforts in Mesoamerica and México.

## 1. Introduction

Malaria has been a major infectious disease in Costa Rica’s history [1,2]. Costa Rica is now among the 21 countries most likely to eliminate malaria by 2020 [3]. To achieve elimination, a robust malaria surveillance system has played a key role in order to understand and reduce malaria transmission [4]. In 1957, Costa Rica created a malaria surveillance and control program (MSCP) following Pan American Health Organization (PAHO) recommendations, and based malaria surveillance on passive case detection by the blood slide examination of patients with malaria symptoms in endemic areas [5,6].

MSCP’s main initial strategy was the systematic use of DDT for indoor residual spraying (IRS) [6]. With this, malaria disappeared from endemic areas across the Pacific basin of Costa Rica [5,6,7]. DDT was replaced by carbamates and pyrethroids in 1986 [6], and IRS use stopped in 1990, except for areas with active transmission [6,7]. Since 2009, IRS has been only deployed focally following the detection of malaria cases [7]. This protocol is still in place [2] to affect a “transmission blockage”, where additional cases are actively searched within 100 m of a diagnosed case, using blood slide examination as a diagnostic, larval peri-domiciliary habitats are treated with larvicides, and three consecutive daily rounds of insecticide thermal fogging using permethrin [1.03% by volume] are applied. In addition, indoor residual spraying with alpha-cypermethrin [0.03 g active ingredient/m^2^] is performed and IRS treatment is repeated every two months over a six-month period [7].

In 1991, Costa Rica was affected by a major earthquake that severely damaged its health services and infrastructure. In the 1990s [8], annual case number increased at least ten-fold. Also, in 1997, 14-day malaria treatment for *Plasmodium vivax* was changed to a five-day radical cure [9], which delivered an insufficient primaquine dose [10]. This treatment policy started to change in 2006 [4], with limited replacement of the five-day radical cure with a pharmacokinetically-sufficient primaquine dose delivered over seven days [1,10]. Previous analyses suggest that this change, coupled with focal mass drug administration (MDA), led to a major shift in malaria transmission in the Huétar Caribe Region (HCR) of Costa Rica [10]. During this time period, for the whole country, these policy changes had a major impact on reducing malaria transmission, despite a two-fold increase in malaria cases during the “Hot” El Niño Southern Oscillation phase [1]. Indeed, MDAs and treatment change were followed by a 33-month hiatus (2013–2015) in nationwide malaria transmission [10].

**Figure A1:**
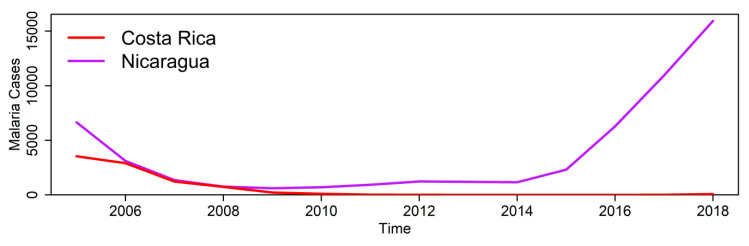
Malaria cases in Costa Rica and Nicaragua between 2005 and 2018. Data are from the Pan American Health Organization (PAHO) [47].

However, starting in 2016, local malaria cases associated with international worker movement, from neighboring Nicaragua, into pineapple production areas [1], and, more recently, into Crucitas, an illegal open-pit gold mining area [11], have led to a resurgence of malaria transmission in the Huétar Norte Region (HNR) of Costa Rica. This situation likely reflects the recent malaria transmission increase in Nicaragua. Despite having a similar trajectory towards malaria elimination from 2005 to 2008, while Costa Rica has achieved a malaria pre-elimination status [3], malaria cases have been exponentially growing in Nicaragua since 2014 (Appendix A Figure A1). In response to a rise in malaria transmission in the HNR during the final months of 2018, the Ministry of Health performed a focal MDA at the end of January 2019. The MDA targeted the population of Boca Arenal, the town most frequently visited by mine workers from Crucitas, and where most locally-transmitted cases have been detected since 2018. Here, we evaluate changes observed in malaria transmission following the implementation of the seven-day treatment in the HNR, which started in 2006, and the MDA in Boca Arenal during 2019.

## 2. Materials and Methods

### 2.1. Surveillance Data

**Figure 1:**
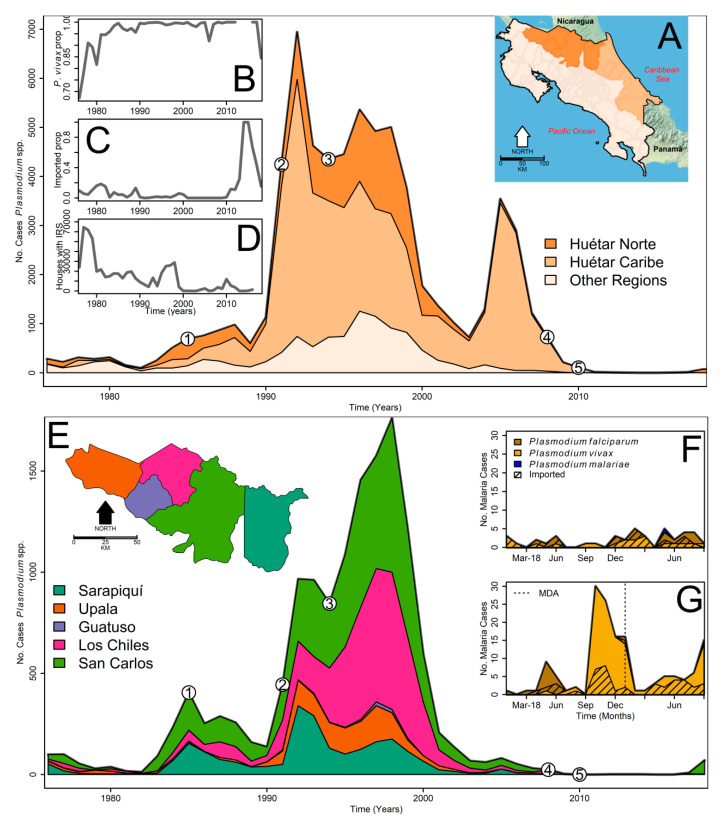
Malaria trends in Costa Rica. (**A**) Annual number of malaria cases reported between 1976 and 2019, highlighting the burden in the Huétar Norte and Huétar Caribe regions. The inset map shows the location of these two regions, whose color codes are presented in the inset legend. The numbered dots represent malaria policy changes and natural catastrophes. Specifically, (1) DDT stopped being used, (2) the Limón earthquake, (3) the implementation of the five-day radical cure, (4) the implementation of the seven-day chloroquine and primaquine treatment, and (5) the implementation of reactive indoor residual spraying (IRS). (**B**) Annual proportion of *Plasmodium vivax* cases in Costa Rica. (**C**) Annual proportion of imported malaria cases in Costa Rica. (**D**) Annual number of houses covered by insecticide residual spraying (IRS) in Costa Rica. (**E**) Annual Number of malaria cases reported in the Huétar Norte region between 1976 and 2019, highlighting the case burden in each one of the five counties comprising the Huétar Norte region. The inset map shows the location of each county, whose color codes are presented in the inset legend. The numbered dots represent the malaria policy changes and natural catastrophes described for panel A. (**F**) Monthly malaria cases, local and imported, in Los Chiles and San Carlos counties from January 2018 to September 2019; the vertical dashed line indicates the application of the mass drug administration (MDA) at Boca Arenal. (**G**) Monthly malaria cases, local and imported, outside Los Chiles and San Carlos counties from January 2018 to September 2019.

We used the annual malaria surveillance data by county, from the Costa Rican Ministry of Health epidemic surveillance database, since 1976. Each case was confirmed by blood slide examination (Figure 1). Malaria case counts were then added by year for the HNR, the HCR, and the rest of the country (Figure 1A). We also computed the proportion of malaria cases due to *Plasmodium vivax* (Figure 1B), the proportion of imported malaria cases (Figure 1C), and the number of households covered by IRS (Figure 1D). We summarized annual malaria case data from 1976 to 2018 for Sarapiquí, Guatuso, Upala, Los Chiles, and San Carlos, the five counties comprising the HNR (Figure 1E). We also compiled monthly imported and local malaria case records from January 2018 to September 2019 in San Carlos and Los Chiles, the two counties with over 80% of the local malaria cases after May 2018, and where the Boca Arenal MDA was done (Figure 1F). For comparison, we compiled the imported and local malaria cases for the rest of the country (Figure 1G). When plotting malaria cases from all of Costa Rica (Figure 1A) and the HNR (Figure 1E), we highlighted the time-points associated with major malaria policy changes and natural catastrophes.

### 2.2. Mass Drug Administration

**Figure 2:**
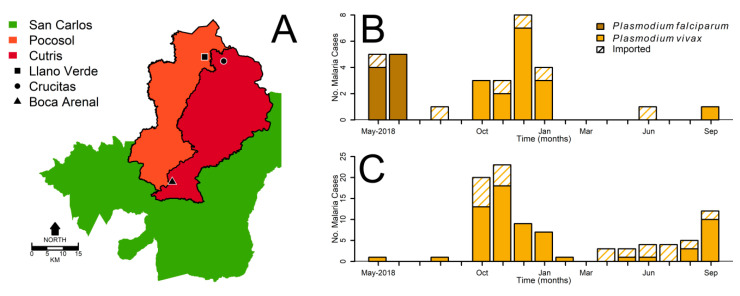
Boca Arenal Mass Drug Administration malaria surveillance (**A**) Map of San Carlos county highlighting the Pocosol and Cutris Districts, the two districts with the largest share of malaria cases in Costa Rica, showing the exact location of Llano Verde, Crucitas, and Boca Arenal. (**B**) The 28-day period malaria cases, local and imported, at Llano Verde, Pocosol District. (**C**) The 28-day period malaria cases, local and imported, at Crucitas and Boca Arenal, Cutris District.

The Boca Arenal MDA was implemented by members from the National Vector Control Management Program (NVCMP), coordinated by Costa Rica’s Ministry of Health Epidemic Surveillance (Vigilancia de la Salud), the coordinating body of the Costa Rican Epidemic Surveillance System (CRESS). Boca Arenal is located in the Cutris district of San Carlos county (Figure 2A). The MDA started by making a census of the 229 buildings of Boca Arenal (BA) town on 26 January 2019.

The census showed that two buildings were not residential, namely, a church only used for religious services and the town’s small grocery. The remaining buildings were residential, but 25 were empty when the MDA was implemented. The people from the remaining 202 households were surveyed for enrollment into the MDA. Households had a mean (±standard deviation (SD)) of 4.2 ± 2.3 residents, ranging from 1 to 14 residents per household. Two households (1% of all households) had all of the residents excluded from the MDA. The remaining 200 households (99%) had at least one member enrolled in the MDA. The households in BA had a total of 866 people; among these, 89 people (10%) were not enrolled in the MDA, because they were either pregnant women (*n* = 6), children below 5 years (*n* = 6), breastfeeding mothers (*n* = 20), breastfeeding children (*n* = 22), or people with other medical conditions. Those with any of these conditions and people who declined to participate comprised a total of 35 people. The MDA started on 28 January 2019 and continued for seven days, with combined “Talamanca” treatment [10] consisting of a 1500 mg total dose of chloroquine administered over three days for adults, with 600 mg delivered on the first day and 450 on the second and third day. Meanwhile, the total primaquine dose was 210 mg total for adults in a 30 mg daily dose for seven days. There was no adjustment of dose by weight, and children between six and twelve years received half of the dose, with people 13 years or older considered to be adults for treatment delivery. The MDA treatment was suspended in case of adverse reactions, which included symptoms of hemolytic anemia, diarrhea, and/or vomiting during the seven-day treatment delivery.

### 2.3. Ethical Clearance

This research was approved and registered by the Consejo Nacional de Investigaciones en Salud (CONIS), in agreement no. 4 from session no.52 held on 11 December 2019, in accordance with article seven from law 9234 for biomedical research involving human subjects in Costa Rica. This study was also declared a sanitary priority by Costa Rica’s Minister of Health on 5 December 2019, in official communication no. MS-DM-8906-2019.

### 2.4. Statistical Analysis

To evaluate the impact of the treatment changes in 2006, we tested for the presence of breakpoints associated with changes in the treatment regimen (i.e., changes in the mean pattern of temporal variability [12]) in the HNR malaria case time series. We used the F-statistic [13] to estimate and test the significance of potential breakpoints, and compared the malaria case number means before and after a breakpoint using Welch’s *t*-test [14], which corrects degrees of freedom to account for unequal variance among compared groups.

For the MDA, we estimated coverage (i.e., how many people were enrolled in the MDA), partial adherence (i.e., how many people received the complete chloroquine part of the treatment), and full adherence (i.e., how many people received the full chloroquine and primaquine doses). We compared differences [14], in the number of people living in households with and without malaria cases prior to the MDA using Welch’s *t*-tests. We also used χ^2^ tests to compare differences in the proportion [14] of people with partial and full MDA adherence between households where malaria cases were previously reported and the rest of the MDA enrolled households at BA.

To evaluate the impact of the MDA on transmission, we estimated the changes over 28-day periods in the time-varying reproduction number [15], *R_t_*, of malaria at BA and Crucitas (Figure 2A). The two locations accounted for 65 of the 135 malaria cases reported from January 2018 to September 2019 in HNR of Costa Rica. BA and Crucitas are linked, as most miners working in Crucitas travel to BA for provisions. To estimate *R_t_*, we first estimated the time from the diagnosis of one case to the next, the serial interval, using data from malaria cases recorded at Llano Verde (LlV; Figure 2B), a rural community close to Crucitas (Figure 2A) and a common stopover for people reaching mining sites. LlV is the location where the first malaria cases were detected in 2018. Unlike the transmission observed at BA and Crucitas (Figure 2C), where transmission seems independent of imported malaria cases, the transmission at LlV was very episodic, with small outbreaks following imported cases (Figure 2B). To estimate the average time between the report of a primary and secondary case, we used the date of symptom onset for the initial imported case and calculated the time to onset for the nearest local cases reported at LlV. When several cases could have served as the source for a local case, we estimated their average as the serial interval. We then used the resulting distribution of serial intervals to generate a posterior sample of serial interval distributions estimated assuming an offset gamma distributed serial interval [16]. This was done employing a Markov Chain Monte Carlo, with a burn-in of 3000 replications, a total of 13,000 replications that were thinned to 1000 final samples. We used the resulting distribution to estimate *R_t_* using a Bayesian framework that considered incidence data, summarized over 28 days, conditional on the serial interval distribution [15]. To ease the interpretation of *R_t_*, we analyzed the cumulative data over 28-day periods, as longer time windows reduce noise in signals through time [17,18].

## 3. Results

**Figure 3:**
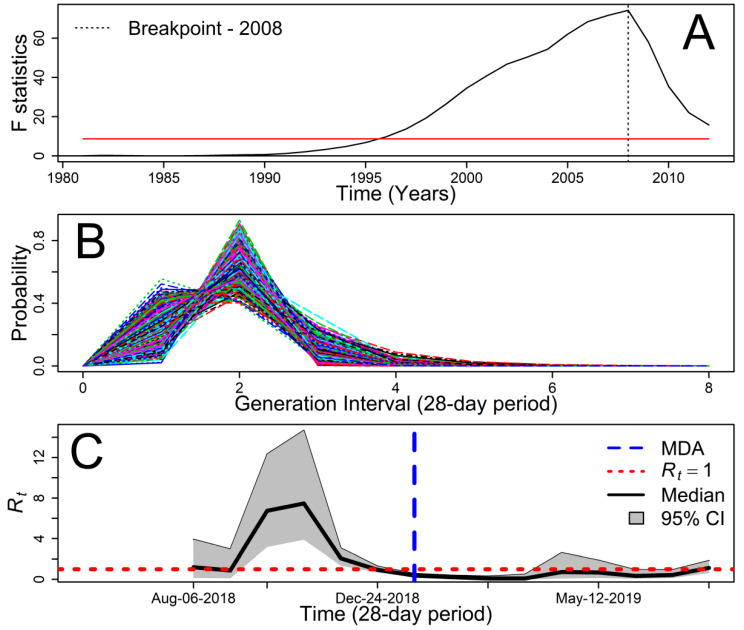
Impact of treatment change policy and the Boca Arenal (BA) Mass Drug Administration (MDA) on malaria transmission (**A**) breakpoint analysis showing the most likely time for a change in malaria transmission, indicated by a vertical dashed line, breakpoints are significant above the red line (*p* < 0.05). (**B**) Posterior sample of malaria serial interval probability distributions generated via Markov Chain Monte Carlo, estimated with data from Llano Verde. Each line represents a simulated serial interval. (**C**) Malaria time-dependent reproduction number *R_t_* at Crucitas and Bocal Arenal. The figure shows the 95% credible intervals (CI) for *R_t_* and the MDA timing at Boca Arenal.

Malaria was always present in the HNR from 1976 to 2009, although the number of cases was very small compared to what was observed in the HCR, the main transmission hotspot in Costa Rica (Figure 1A). From 1976 to 2018, most cases were due to *Plasmodium vivax* (Figure 1B), and with the start of the pre-elimination period in 2013, most of the reported cases were imported. In 2018 and 2019 the trend reversed, with more local than imported cases (Figure 1C). IRS (Figure 1D) probably played a major role in reducing malaria cases until the late 1970s, but afterwards played a reduced role, not being significantly associated (Pearson′s *r* = −0.06, *t* = −0.41, d.f. = 40, *p*-value = 0.68) with the number of cases. Malaria transmission decreased following changes in treatment and focalized MDA in the HCR (Figure 1A). In the HNR, transmission dropped following the change from the five-day radical cure to the seven-day treatment (Figure 1E) in 2008 (Figure 3A), where the annual mean malaria cases (±SD) significantly (Welch′s *t* = 4.5, d.f. = 32.381, *p*-value = 6.8 × 10^−5^) decreased from 415 ± 511 during 1976–2008 to 8 ± 22 during 2009–2019.

**Figure A2:**
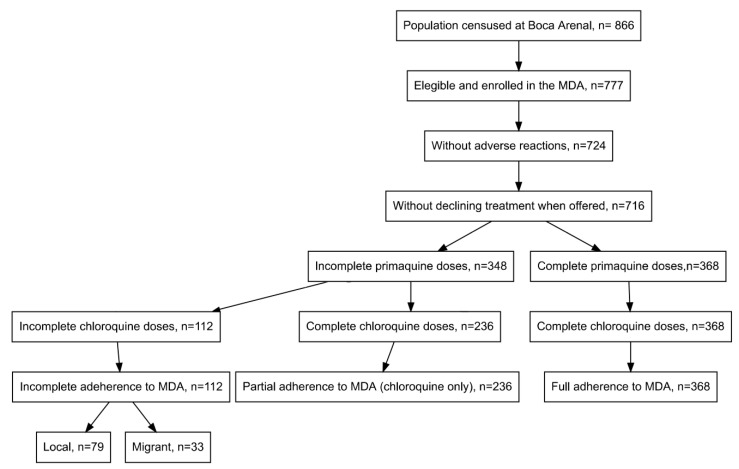
Flowchart for MDA individual recruitment in Boca Arenal, Región Huétar Norte, Costa Rica between January to February 2019.

The MDA had a coverage of 90%, enrolling 777 of the 866 people living in BA; 48% (*n* = 368) received a full treatment dose, and 236 people (30% of the enrolled in the MDA) received the full chloroquine dose and incomplete primaquine doses. The 22% (*n* = 173) of people that did not receive all of the chloroquine doses included eight that declined to continue the treatment when visited by personnel from the PNMV and 53 people whose treatment was suspended after showing adverse reactions. None of the adverse reactions included symptoms associated with primaquine-induced severe hemolytic anemia. The remaining 112 people were absent more than four times during the PNMV visits. This group included 33 people who were migrant laborers who left Boca Arenal during the MDA, according to other household members. A flowchart describing the MDA is presented in Appendix A Figure A2 .

When considering the prior history of malaria in the Boca Arenal households where the MDA was implemented, we found that households (*n* = 13) with a history of malaria cases during 2018 and 2019 had a similar (Welch’s *t* = 0.63824, d.f. = 14.10, *p*-value = 0.534) number of residents enrolled in the MDA (mean ± S.D. = 4.23 ± 2.01, range = 1–8) as the households (*n* = 187) without malaria (3.86 ± 2.21, range = 1–12), the difference not being statistically significant. We also found that the percentage adherence to the full chloroquine and primaquine treatment was slightly higher in households with a recent history of malaria cases (56 ± 41, range = 0–100%) than in households without cases (45 ± 40, range = 0–100%), the difference not being statistically significant (χ^2^ = 0.165, d.f. = 1, *p*-value = 0.6844). The percentage adherence to the full chloroquine treatment was very high in both groups. In households with former malaria cases, it was 86 ± 24 (range = 25–100%), and in households without former malaria cases, it was 75 ± 33.02 (range = 0–100%), the difference not being statistically significant (χ^2^ = 0.06, d.f. = 1, *p*-value = 0.802).

The serial interval distribution, i.e., the time it takes for a case to generate a new case via vector transmission [19], for LlV had a mean of 51 days (95% CI: 44–57) with an SD of 35 days (95% CI: 33–40), which was roughly approximate to a mean (±SD) of 1.81 ± 1.26 for 28-day periods (Figure 3B). Prior to the MDA, the *R_t_* in BA–Crucitas already started to decrease (Figure 3C), but it decreased further, at least until May 2019, when the mean *R_t_* value started to grow (Figure 3C). After May 2019, the *R_t_* was always below one, although with credible intervals not different from one. This result suggests that the impacts of the MDA lasted between three and five months, considering the MDA was done in late January 2019. Indeed, the mean (±SD) *R_t_* for June 2018 to January 2019 was 1.97 ± 0.39, while for February–May of 2019 it was 0.243 ± 0.078, significantly below 1; however, starting with the re-introduction of imported cases in May 2019, *R_t_* had a mean value of 1.00 ± 0.26. Finally, it is worth highlighting that the malaria cases between May and September 2019 occurred in people that had neither full nor partial (full chloroquine) adherence to the MDA treatment in BA, and that from May 2018 to September 2019, only one case was a *P. vivax* relapse—a migrant worker not enrolled in the MDA, who did not take the full 7DCPT the first time he was attended by the Costa Rican universal health care system.

## 4. Discussion

Our analysis suggests that both the treatment change that started in 2006 and the BA–Crucitas MDA of 2019 were associated with malaria transmission reduction in the HNR. In both cases, these changes accompanied a standard malaria elimination protocol in place since 2009 [2], the “transmission blockage” described in the introduction, which is based on reactive human case surveillance coupled with supervised treatments and vector control, following the detection of locally transmitted malaria cases. Although this blockage can immediately reduce transmission, our results suggest that eliminating infection reservoirs with appropriate treatments and focalized MDAs may play a critical role for malaria elimination and can fill an important gap in reducing transmission, considering that the dominant malaria vector species in Mesoamerica and Mexico, *Anopheles albimanus*, is mainly exophilic and exophagic [20,21,22]. IRS thus may have limited impacts on its ability to reduce malaria transmission. For example, the “transmission blockage” protocol has been used in Costa Rica since 2002, where its use in the HCR did not help reduce repeated transmission in the same households of Talamanca county, a milestone only achieved after the implementation of MDAs and case management based on seven-day treatments [7,10]. In other words, vector control and other tools aimed at reducing human–vector contact, although able to reduce transmission, might not be enough to eliminate malaria in Costa Rica and similar settings in Mesoamerica and Mexico.

The BA MDA had a full adherence of nearly 50% of the BA population, helping to keep *R_t_* below one in BA and Crucitas. Local transmission was interrupted for at least four months, despite ongoing malaria transmission elsewhere in the HNR, and also in Limón (part of the HCR) and Chomes de Puntarenas (on the Pacific Coast) [23]. However, focal MDAs in Costa Rica can be made more effective. Currently, Costa Rica follows PAHO/WHO recommendations [24] to exclude breastfeeding women from malaria treatments, despite clinical evidence that suggests it is safe for this subpopulation and their breastfeeding children [25]. Thus, the 90% coverage of the BA MDA that we observed could be increased by at least 2% if breastfeeding mothers are included. Adherence can be increased if additional resources are included to follow-up the individuals that remained in the area but were not present when the supervised treatment was delivered, potentially increasing adherence by at least 10% and up to 40%, which was not possible when the 2019 BA MDA was implemented because of the current structural macro-economic adjustments in Costa Rica [26]. To increase adherence, the introduction of tafenoquine, a single-dose malaria drug, could dramatically increase treatment adherence, as only one dose would be necessary to treat key populations [27], like migrant workers that left BA during the MDA and would not benefit from follow-ups, to people that missed MDA doses by being absent during the PNMV visits to administer the 7DCPT. Migrants are a major threat to elimination in Costa Rica, but also regionally in Mesoamerica and Mexico, because the poor treatment adherence can become a selective pressure for drug resistance [28], and their high mobility accelerates the spread of parasites [29]. However, before implementing tafenoquine use, the CRESS needs to implement reliable field tests for G6PD [30] deficiency, in order to avoid adverse reactions in subjects likely to suffer hemolytic anemia. Something else that could be refined is MDA deployment timing, frequency, and coverage, where the high-quality data generated by the CRESS could be used with mathematical models [31] to optimize these aspects in the field implementation of MDAs.

Evidence from Latin America [32] and other settings [33,34,35] suggests that MDAs and proper curative treatments are critical and effective to eliminate malaria when transmission is very focal [28], and might even have a population-wide anti-malaria protective impact [36]. In Costa Rica, the 7DCPT includes primaquine, because this drug targets *P. vivax* hypnozoites, dormant forms responsible for malaria relapses [7]. However, primaquine can be used in low doses to kill gametocytes of *Plasmodium* spp. that do not cause relapses [37]. For example, single primaquine doses have been used for their gametocidal effects to treat *P. falciparum* and *P. malariae* [38]. Moreover, malaria treatments with primaquine have been shown to reduce malaria case burden in low-transmission settings [38]. To improve the current malaria surveillance, the CRESS needs to improve the diagnosis of inapparent infections [39] and gametocytemic loads [40], but also mosquito surveillance in hotspots. To date, we suspect that *An. albimanus* is the main malaria vector in Cutris and Pocosol districts, but we have been unable to confirm this, because entomological surveillance is currently restricted to mosquito larvae, and the available distribution records for this species and other *Anopheles* spp. in Costa Rica are not current [6,41]. However, expanding entomological surveillance to adult mosquitoes and testing them for infections could be used as an early warning to trigger reactive case detection, to deploy timely vector control interventions, or even to forecast infection based on weather records and remotely-sensed information collected by satellites [42,43]. The CRESS also needs to develop capacities to routinely genotype circulating malaria parasites in humans and mosquitoes, as such information will allow for a better understanding of the propagation of malaria, to recognize whether outbreaks have a clonal structure [44], arising drug resistance, and to better understand the geographic source of parasites in order to coordinate regional efforts for malaria elimination in Mesoamerica and Mexico. This region, as a low-transmission setting, is highly heterogeneous [45], and needs to develop innovative interventions and tools for malaria elimination surveillance that consider parasite–vector bionomics and transmission in key population groups, including migrant workers, a population often comprising malaria index cases in malaria outbreaks in Costa Rica, and, as shown here, with problems achieving full adherence to 7DCPT in focal MDAs, because of their increased mobility and working conditions.

## 5. Conclusions

We are aware that beyond the specific actions to eliminate malaria described here, a wider desirable goal is improving the quality of life in communities currently affected by malaria in Costa Rica and elsewhere in Mesoamerica and Mexico. The Pocosol and Cutris Districts, where malaria has the largest case burden in Costa Rica, are in the lowest 10th percentile of human development in Costa Rica [46]. These districts have some of the worst coverage of public services [46] and are at risk of being afflicted by other infectious diseases associated with poverty. The lack of socio-economic development alternatives in these poor and disadvantaged districts of Costa Rica has led to the emergence of illegal open pit gold mining in Crucitas. Poverty has thus led to a serious environmental problem [11] that has now become a major threat to malaria elimination in Costa Rica.
